# Improved Cancer Detection Using Artificial Intelligence: a Retrospective Evaluation of Missed Cancers on Mammography

**DOI:** 10.1007/s10278-019-00192-5

**Published:** 2019-04-22

**Authors:** Alyssa T. Watanabe, Vivian Lim, Hoanh X. Vu, Richard Chim, Eric Weise, Jenna Liu, William G. Bradley, Christopher E. Comstock

**Affiliations:** 1 Keck School of Medicine, University of Southern California, 1975 Zonal Avenue, Los Angeles, CA 90033 USA; 2 Keck School of Medicine, University of Southern California, 2711 North Sepulveda Boulevard, Suite 284, Manhattan Beach, CA 90266-2725 USA; 3 School of Medicine, Koman Family Outpatient Pavilion, University of California San Diego, 9400 Campus Point Drive, La Jolla, San Diego, CA 92037 USA; 4 CureMetrix Incorporated, 9404 Genesee Avenue, Suite 330, La Jolla, San Diego, CA 92037 USA; 5 Sharp Grossmont Hospital, 5555 Grossmont Center Drive, La Mesa, CA 91942 USA; 6 School of Medicine, University of California, San Diego, 9500 Gilman Drive, Suite 120, La Jolla, San Diego, CA 92093 USA; 7 Memorial Sloan Kettering Cancer Center, 300 East 66th Street, New York, NY 10065 USA

**Keywords:** Mammography, Artificial intelligence, Computer-aided detection, Cancer detection, Breast cancer, Deep learning

## Abstract

To determine whether cmAssist™, an artificial intelligence-based computer-aided detection (AI-CAD) algorithm, can be used to improve radiologists’ sensitivity in breast cancer screening and detection. A blinded retrospective study was performed with a panel of seven radiologists using a cancer-enriched data set from 122 patients that included 90 false-negative mammograms obtained up to 5.8 years prior to diagnosis and 32 BIRADS 1 and 2 patients with a 2-year follow-up of negative diagnosis. The mammograms were performed between February 7, 2008 (earliest) and January 8, 2016 (latest), and were all originally interpreted as negative in conjunction with R2 ImageChecker CAD, version 10.0. In this study, the readers analyzed the 122 studies before and after review of cmAssist™, an AI-CAD software for mammography. The statistical significance of our findings was evaluated using Student’s *t* test and bootstrap statistical analysis. There was a substantial and significant improvement in radiologist accuracy with use of cmAssist, as demonstrated in the 7.2% increase in the area-under-the-curve (AUC) of the receiver operating characteristic (ROC) curve with two-sided *p* value < 0.01 for the reader group. All radiologists showed a significant improvement in their cancer detection rate (CDR) with the use of cmAssist (two-sided *p* value = 0.030, confidence interval = 95%). The readers detected between 25 and 71% (mean 51%) of the early cancers without assistance. With cmAssist, the overall reader CDR was 41 to 76% (mean 62%). The percentage increase in CDR for the reader panel was significant, ranging from 6 to 64% (mean 27%) with the use of cmAssist. There was less than 1% increase in the readers’ false-positive recalls with use of cmAssist. With the use of cmAssist TM, there was a substantial and statistically significant improvement in radiologists’ accuracy and sensitivity for detection of cancers that were originally missed. The percentage increase in CDR for the radiologists in the reader panel ranged from 6 to 64% (mean 27%) with the use of cmAssist, with negligible increase in false-positive recalls.

**Electronic supplementary material:**

The online version of this article (10.1007/s10278-019-00192-5) contains supplementary material, which is available to authorized users.

## Introduction

Mammography is a widely accepted tool for breast cancer screening [1, 2]. About 50% of mammographically detected breast cancers are visible retrospectively on prior studies [3]. Many of these cancers are obscured by dense breast tissue, subtle on mammography, or missed through human error. In addition, there exists a high percentage of false-positive mammography results and unnecessary biopsies [4]. For these reasons, the need exists for methods and techniques that can improve sensitivity and specificity in mammography interpretation.

Double reading of mammograms has been shown to increase the sensitivity of screening mammography when compared to single reading by 5–15%, and this practice is still used in Europe today. In the USA, computer-aided detection (CAD) is used for more than 70% of screening exams [5]. However, despite initial promise [5–7], the overall benefit of currently available CAD remains controversial. One of the most comprehensive retrospective studies to date on the efficacy of CAD was conducted in 2015 [8] and included 271 radiologists across 66 facilities and more than 300,000 patients. The study found no positive benefit on radiologists’ performance from CAD assistance. On the contrary, the study showed significantly decreased sensitivity of radiologists with CAD (odds ratio, 0.53; 95% confidence interval = [0.29, 0.97]) and an overall increase in unnecessary callbacks.

Early detection analysis—the study of mammograms taken more than 9 months before a cancer diagnosis—has not been as widely studied. In 2000, Warren Burhenne et al. [3] reported a 27% reader sensitivity in detecting cancer in prior mammograms without CAD, but did not evaluate the effect of CAD on reader sensitivity. More recently, the rise of deep learning technology from the field of artificial intelligence (AI) has led to a new generation of algorithms for image analysis. To the authors’ knowledge, the clinical impact of using AI-CAD in mammography has not been validated in the published literature. The retrospective early detection reader study presented in this paper explores the potential benefit of AI-CAD using cmAssist™ (an AI-CAD software for mammography recently developed by CureMetrix, Inc., La Jolla, CA) to enhance the ability of readers with varying skill levels to detect cancers in an archive of false-negative mammograms obtained up to 5.8 years prior to the eventual recall and workup for breast cancer. Specifically, this study determines the efficacy of cmAssist in improving radiologists’ sensitivity in breast cancer screening and detection using a challenging set of cancer cases that were originally missed using R2 ImageChecker CAD.

## Materials and Methods

Financial support was provided by CureMetrix, Inc. for this study. All authors had control of the data and information submitted for publication. Because this was a retrospective study of patients’ clinical records, a waiver was obtained for Human Study IRB approval. This study has been performed in accordance with the ethical standards as laid down in the 1964 Declaration of Helsinki and its later amendments. For this type of study, formal consent is not required. All mammograms were anonymized using a Health Insurance Portability and Accountability Act (HIPAA)-compliant protocol.

### Subject Group

A set of 2D Full-Field Digital Mammograms (FFDM) was collected from a community healthcare facility in Southern California for retrospective study. The mammograms were originally interpreted by community-based radiologists using the R2 ImageChecker CAD, version 10.0 (Hologic, Inc., Sunnyvale, CA). All patients in the collected dataset were females, aged 40–90 years, who had a biopsy performed between October 2011 and March 2017. Of 1393 patients, 499 had a cancer biopsy, 973 had a benign biopsy, and 79 had both cancer and benign biopsies. None of these cases were used as training data for the cmAssist algorithm, and all the cases were quarantined for the purposes of this study.

For this study, authors define a prior mammogram as one taken more than 270 days (9 months) before a tissue biopsy was performed. Of the 499 patients having biopsy-confirmed breast cancer, 317 had prior mammograms. Of the cancer patients with prior mammograms, there were 139 patients who had retrospective findings on their prior mammograms.

Prior mammograms that showed retrospective findings were marked by two Mammography Quality Standards Act (MQSA)-certified “validating radiologists” (A.T.W., V.L.) who had current mammograms and biopsy results available for reference. The markings were archived along with a written recording of location, lesion type, and tissue density. In cases of conflict, cases were reviewed again and a consensus was obtained in categorizing those lesions. These truth markings were hidden from the subsequent blinded panel of radiologists, but used for correlation of the recall results. The validating radiologists were excluded from the reader study.

The validating radiologists categorized the retrospective findings on prior mammograms into one of the following categories:

- Actionable—the lesion that eventually was biopsied was recallable in a clinical setting,
- Non-actionable—the lesion that was eventually biopsied was subthreshold for recall,
- Excluded—ipsilateral prior lumpectomy and synthesized or three-dimensional tomography images.

The cmAssist AI-CAD was considered to have correctly marked the Actionable lesion if a cmAssist marking overlapped the markings made by the validating radiologists.

A total of 155 prior mammograms from 90 patients were deemed Actionable. The reader study was restricted to the oldest Actionable prior mammogram per patient, termed “Earliest Actionable.” The Earliest Actionable prior mammograms were chosen to make the data set as rigorous as possible, and to create a reasonable number of cases for a single sitting. The Earliest Actionable prior mammograms consisted of women with a mean age of 65.4 years (age range, 40–90 years) at diagnosis. The Earliest Actionable prior mammograms were obtained between 0.76 and 5.8 years (mean, 2.1 years) prior to the current mammogram.

An additional 32 normal studies were included in the reader study to reduce reader bias. These patients were confirmed to have 2 or more years of subsequent normal mammograms.

Time-aggregate data from 1/1/2009 through 12/31/2016 (for the institution from which mammographic studies were collected for the study presented in this paper) reveals a cancer detection rate (CDR) of 4.5 per thousand (108,698 mammography cases, 488 malignant biopsy cases), compared to the US national average CDR of 5.1 per thousand reported by the Breast Cancer Surveillance Consortium for the period 2007–2013 [9]. Therefore, the cancers missed by the original interpreting radiologists (who had the benefit of R2 ImageChecker CAD) do not reflect lack of skill, but instead represents a set of cancers that could be missed in daily practice due to the inherent complexity of mammography interpretation.

### Brief Description of cmAssist

cmAssist is a recently developed AI-CAD for mammography which incorporates a popular form of artificial intelligence called deep learning. cmAssist was trained using curated mammograms from multiple institutions consisting of biopsy-proven benign and malignant lesions, as well as validated normal mammograms (BIRADS 1 and 2 studies with at least 2-year follow-up of negative diagnosis). None of the mammograms that were evaluated in this retrospective study were used in the development of cmAssist. The cmAssist algorithm is based on multiple custom deep learning-based networks that work together to achieve high sensitivity without sacrificing specificity. Furthermore, the training of the algorithms utilizes a proprietary, patent-pending data augmentation technique to enrich the different presentations of cancer and benign structures in our training set. The training set is comprised of images acquired on multiple different makes and models of mammography units. The software is vendor agnostic, runs on for presentation images (which means that raw DICOM is not needed) and no calibration is needed, which are advantages over traditional CAD.

**Fig. 1:**
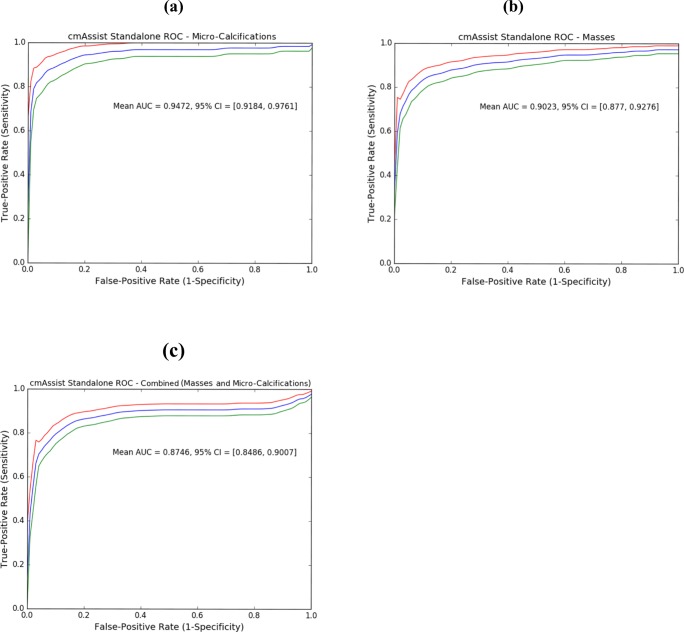
cmAssist AI-CAD stand-alone ROC on CureMetrix quarantined test dataset. cmAssist’s stand-alone efficacy, based on the CureMetrix quarantined test dataset consisting of 836 normal studies, 126 biopsy-proven micro-calcification cancer studies (**a**), 323 biopsy-proven mass cancer studies (**b**), achieves an AUC of 0.947 (95% CI = [0.918, 0.976]) and of 0.902 (95% CI = [0.877, 0.928]) for micro-calcifications and masses, respectively. The combined ROC (**c**) corresponds to an overall AUC of 0.875 (95% CI = [0.849, 0.901]). AI-CAD, artificial intelligence-based computer-aided detection; CC (CC view only), ‘cranial-caudal’ view; CDR; cancer detection rate; cmAssist, prototype AI-CAD software from CureMetrix, Inc.; LCC, the left ‘cranial-caudal’; LMLO, the left ‘Mediolateral-Oblique’; neuScore, cmAssist quantitative score results (scale of 0–100); RCC, the right ‘cranial-caudal’; RMLO, the left ‘mediolateral-oblique’

The cmAssist’s stand-alone efficacy, as measured by the traditional receiver operating characteristic (ROC) curve, is shown in Fig. 1 for masses, micro-calcifications, and for all lesions. The stand-alone efficacy is based on the CureMetrix internal test dataset, which consists of 836 normal studies (3348 images), 323 biopsy-proven mass cancer studies, and 126 biopsy-proven micro-calcifications cancer studies. The test dataset is quarantined and has never been used in any aspect or any phase of the development of cmAssist.

### Reader Study Procedure

A blinded reader study was performed to assess the potential clinical benefit of cmAssist for improved accuracy in cancer detection by mammographers. The enriched data set comprises of 90 false-negative Earliest Actionable prior mammograms and 32 normal exams, for a total of 122 exams.

Seven MQSA-certified radiologists of various training and experience levels were recruited for the reader panel. The reader study was performed using a high-resolution viewing workstation and FDA-approved mammography viewing software (WorkStation One™, Three Palm Software, Carmel, CA) in a reading room that met MQSA standards.

The cases were shown to each radiologist without any clinical information or comparison to prior studies. The readers were informed that there was a combination of normal and recallable mammograms. The cases were put into a randomly ordered worklist and displayed to each radiologist in the same order by an independent proctor. Each radiologist was asked to view each mammogram without cmAssist markings and make a clinical decision about recall. Subsequently, each radiologist was provided with the cmAssist markings and their corresponding quantitative scores (neuScore™, scale of 0–100) and given the opportunity to change the clinical decision, i.e., the reader has the option to add/remove areas of recall based on the review of the cmAssist results. The proctor recorded location and lesion type for recalls and whether the reader changed recommendation after cmAssist review.

The change in CDR and false-positive recall rate (after cmAssist compared to before cmAssist) were calculated for each radiologist. Statistical significance was calculated using the two-sided Student’s *t* test with a null hypothesis of a mean value of zero. The data was also analyzed based on lesion type (mass versus micro-calcifications) and tissue density. If the radiologist recalled the patient, but indicated the incorrect quadrant or incorrect laterality, it was considered a false-negative recall.

The overall reader group accuracy before and after cmAssist was evaluated using ROC and AUC analysis. The statistical significance of the change in AUC was established using the bootstrapping method [10] with 10,000 samples to establish the two-sided 99% confidence interval (CI), and by showing that the null hypothesis (change in AUC = 0%) is outside of our two-sided 99% CI, i.e., two-sided *p* < 0.01.

## Results

### Subject Group

Of the 317 cancer patients with prior mammograms, 44% of the patients (*n* = 139) had retrospective findings and the remaining 56% of patients (*n* = 178) had de novo cancers, with no retrospective findings seen on their prior mammograms. Out of the patients who had retrospective findings, 90 of the 139 patients (65%) were deemed Actionable, 40 were deemed non-Actionable, and 9 were excluded (see “Materials and Methods” section). For brevity, the authors refer to all cases with micro-calcifications as the leading lesion type as calcifications and all remaining cases, such as focal asymmetry or mass with micro-calcifications, as mass. The Actionable prior mammogram cases consisted of 17 micro-calcification cases and 73 mass cases (Supplementary Table 1). The tissue density breakdown of the 90 cancer cases is as follows: fatty (*n* = 4, 4%), scattered (*n* = 43, 48%), heterogeneously dense (*n* = 37, 41%), and extremely dense (*n* = 6, 7%).

### Reader Study

All readers in the panel are American Board of Radiology-certified radiologists with MQSA certification. The individual reader experience and training is described as follows:

- Readers 1 and 2 are general radiologists with less than 5 years of practice experience.
- Reader 3 is an experienced general radiologist with 42 years of experience.
- Readers 4, 5, and 6 are mammography fellowship-trained radiologists.
- Reader 7 is a general radiologist with 19 years of practice experience.

The influence of cmAssist on true-positive recalls was measured through an analysis of the 90 biopsy-proven cancer cases in the data set as shown in Table 1. As summarized in Table 1, there was improvement in CDRs with the use of cmAssist for all radiologists in this study regardless of their level of training and experience, with an average increase in CDR of 11% (range 4–26%). There was more benefit seen with the less-experienced general radiologists (Readers 1 and 2) than for the mammography fellowship-trained radiologists (Readers 4–6). The overall reader CDR without cmAssist was 25 to 71% (mean = 51%). With cmAssist, the overall reader CDR was 41 to 75% (mean = 62%). The mean percentage increase in the CDR with assistance of cmAssist was 6 to 64% (mean = 27%).

**Table 1:** Actionable Lesions on False Negative Mammograms The distribution of Actionable lesions in the cancer group classified by the validating radiologists

Lesion Type	Count
Mass	50
Microcalcifications	16
Mass and Microcalcifications	9
Architectural Distortions	5
Mass and Architectural Distortions	4
Asymmetry	3
Architectural Distortion and Microcalcifications	1
Microcalcifications and Asymmetry	1
Focal Asymmetry	1
Total	90

Based on study results, some trends were seen from the reader panel; those most influenced by cmAssist (Group 1: Readers 1–3) and those less influenced (Group 2: Readers 4–7). Group 1 included the two less-experienced radiologists (Readers 1 and 2) and one general radiologist who had been trained over 4 decades prior to this study. These readers appeared to have high reliance on cmAssist, with reversal from no recall to recall in a significant number of the missed cancers with cmAssist assistance. One of the general radiologists (Reader 3) showed dramatic benefit (percent increase of 64% in CDR) with cmAssist assistance. Reader 1, a less-experienced general radiologist with less than 3 years in practice, had a CDR percent increase of 61% after reviewing with cmAssist assistance. As expected, the mammography fellowship-trained mammographers (Group 2) had higher CDRs than the less-experienced, general radiologists (Group 1) without cmAssist. One striking observation is that cmAssist assistance brought the sensitivity of the two least-experienced general radiologists (Group 1: Readers 1 and 2) to CDRs that exceeded the CDR of 75% of group 2 readers.

The influence of the cmAssist on false-positive recalls was measured through an analysis of the 32 normal cases in the data set as shown in Table 2. As summarized in Table 2, the change in false-positive recall rates varied from − 6 to + 6%. For three of the readers, there was no increase in false-positive recalls, and for one reader, there was a reduction in false-positive recalls. Overall, the false-positive recall rate increased by less than 1% with the use of cmAssist (mean = 0.89%). The increase in false-positive recalls was only seen in group 1 (less-experienced, general radiologists). Among readers of group 2, there was a 6.25% decrease in the false-positive recall rate for one reader, with no change in the false-positive recall rates for the remaining readers.

**Table 2:** Effect of AI-CAD on True Positive Recall Reader sensitivity (cancer detection rate) in prior mammograms with Actionable findings before and after review of AI-CAD shows improvement in CDR for all of the readers

Radiologist	Years of experience	Cancer Detection Rate before AI-CAD	Cancer Detection Rate after AI-CAD	Increase in Cancer Detection Rate after AI-CAD	Percentage Change in Cancer Detection Rate
1	3	42%	68%	26%	62%
2	3	54%	68%	14%	26%
3	42	25%	41%	16%	64%
4	5	46%	53%	7%	15%
5	6	71%	75%	4%	6%
6	3	56%	60%	4%	7%
7	19	61%	67%	6%	10%
Average		51%	62%	11%	27%

The cmAssist AI-CAD showed benefit as a decision support tool about equally for masses and calcifications, as indicated in Table 3 where a summary of the readers’ recall decision-making for micro-calcifications versus masses are summarized. The less-experienced readers were influenced to recall more actionable mass lesions than the experienced readers.

**Table 3:** Effect of AI-CAD on False Positive Recall. Presents the false positive recall rates based on the normal cases in the data set

Radiologist	False positive recalls before AI-CAD	Increase in false positive recalls after AI-CAD	Reduction of false positive recalls after AI-CAD	False positive recalls after AI-CAD	Change in false positive Recall Rate (%)
1	7	4	-3	8	3%
2	6	2	0	8	6%
3	4	2	-1	5	3%
4	8	0	-2	6	-6%
5	6	0	0	6	0%
6	9	0	0	9	0%
7	9	0	0	9	0%
Average	7.0	1.1	-0.9	7.3	<1%

cmAssist appears to be beneficial across all tissue densities. The increased reader CDR with the assistance of cmAssist is shown to be statistically significant in scattered (*p* value = 0.026) and heterogeneously dense (*p* value = 0.061) cases. The reader CDR for fatty and extremely dense mammograms also appear to be benefited by cmAssist, but statistical significance was not demonstrated.

**Fig. 2:**
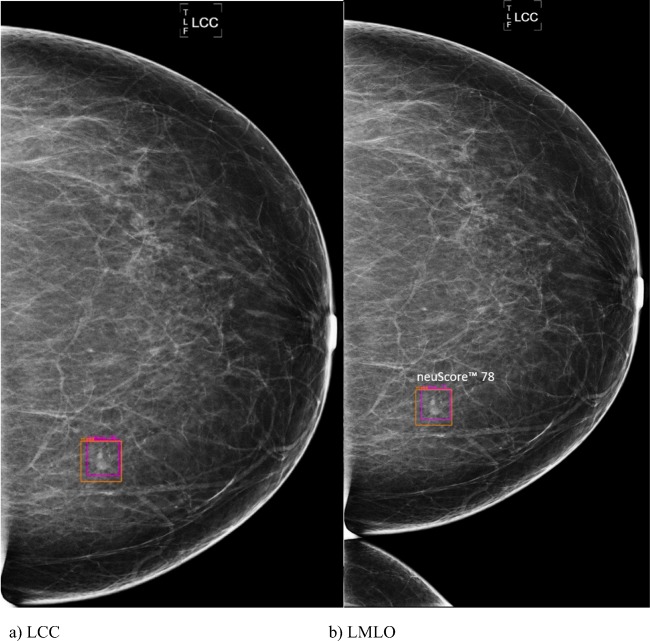
**a**, **b** Mammogram of 63-year-old woman. The LCC (left cranial-caudal, **a**) and LMLO (left mediolateral oblique, **b**) views show missed small spiculated cancer in a fatty breast detected by cmAssist 1140 days (more than 3 years) prior to diagnosis (neuScore = 78). The magenta box is the cmAssist flag on the CC view only. The retrospective cancer as marked by the validating radiologists in the orange boxes (ground truth). This case was initially flagged by one radiologist but recalled by four more after viewing the cmAssist analysis. AI-CAD, artificial intelligence-based computer-aided detection; CC (CC view only), ‘cranial-caudal’ view; CDR; cancer detection rate; cmAssist, prototype AI-CAD software from CureMetrix, Inc.; LCC, the left ‘cranial-caudal’; LMLO, the left ‘Mediolateral-Oblique’; neuScore, cmAssist quantitative score results (scale of 0–100); RCC, the right ‘cranial-caudal’; RMLO, the left ‘mediolateral-oblique’

The benefit of AI-CAD in a fatty breast (tissue density = 1) is presented in Fig. 2 a and b where only one of the readers out of seven recalled the small cancer in a fatty breast. With AI-CAD assistance, an additional four readers correctly changed their decision to recall that patient. It is noted parenthetically that there is a lesion that appears as two small adjacent circumscribed masses in MLO view (Fig. 2b), superior to the ground truth. cmAssist scored this lesion as subthreshold, and a review of the mammograms taken 1 year after those in Figs. 2 shows that the lesion in question is in fact stable.

**Fig. 3:**
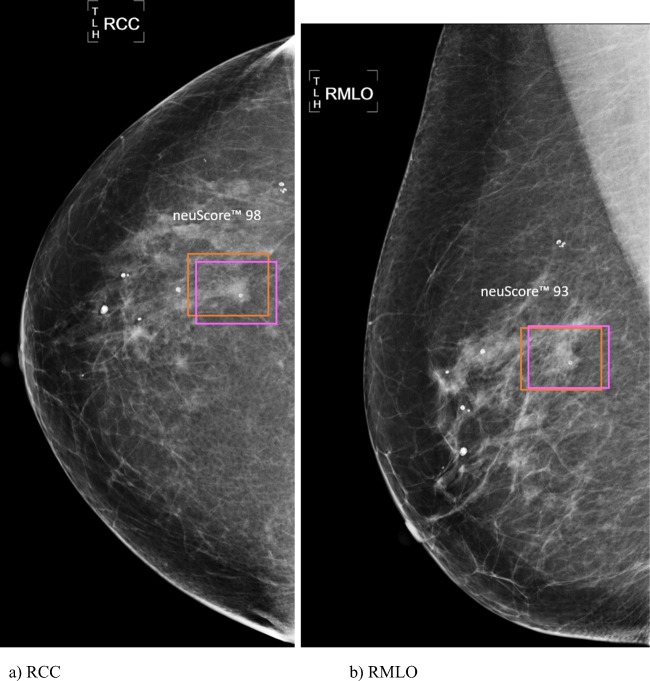
**a**, **b** Mammogram of 78-year-old woman. RCC (cranial-caudal, **a**) and RMLO (right mediolateral oblique, **b**) mammograms show a missed cancer detected by AI-CAD 783 days (more than 2 years) prior to diagnosis (neuScore = 98). This case was initially marked for recall by five of seven radiologists without cmAssist. After reviewing the cmAssist markings, the remaining two radiologists converted to recall. The magenta cmAssist box corresponds with the validated Actionable finding (missed cancer) that is marked in the orange (truth) box. AI-CAD, artificial intelligence-based computer-aided detection; CC (CC view only), ‘cranial-caudal’ view; CDR; cancer detection rate; cmAssist, prototype AI-CAD software from CureMetrix, Inc.; LCC, the left ‘cranial-caudal’; LMLO, the left ‘Mediolateral-Oblique’; neuScore, cmAssist quantitative score results (scale of 0–100); RCC, the right ‘cranial-caudal’; RMLO, the left ‘mediolateral-oblique’

An example of a missed cancer in a patient with scattered densities (tissue density = 2) is depicted in Fig. 3 a and b. Five of the seven readers flagged the cancer independently, but the cmAssist flag influenced the two remaining readers to change from no recall to recall. Table 4 provides a summary of reader recall decision-making for each of the four breast density levels.

**Table 4:** **a** Reader panel decision making for calcifications. The readers recalled an average of 3.4 additional cancerous calcifications with benefit of AI-CAD. **b** Reader panel decision making for masses. Readers recalled an average of 6.4 additional cases of malignant masses with AI-CAD assistance

**a**				
Radiologist	Years of experience	Calc cases recalled without AI-CAD	Additional calc cases recalled with AI-CAD	Calc cases ignored with AI-CAD
1	3	8	6	3
2	3	9	3	5
3	42	1	8	8
4	5	7	1	9
5	6	14	1	2
6	3	5	2	10
7	19	8	3	6
Average		7.4	3.4	6.1
**b**				
Radiologist	Years of experience	Mass cases recalled before AI-CAD	Additional mass cases recalled after AI-CAD	Flagged mass cases ignored by readers
1	3	30	17	8
2	3	40	10	9
3	42	22	6	22
4	5	34	5	16
5	6	50	3	10
6	6	45	2	8
7	19	47	2	7
Average		38	6.4	11

**Fig. 4:**
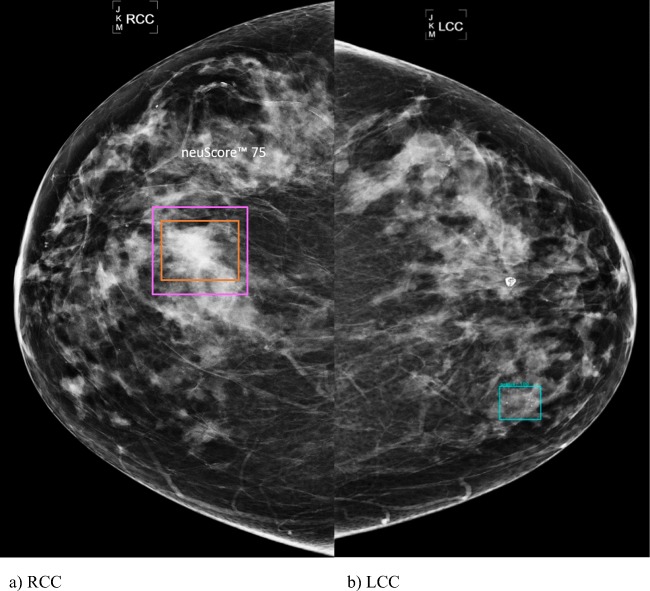
**a**, **b** Mammograms of 78-year-old woman. RCC prior mammograms, showing a missed cancer detected by AI-CAD 400 days (more than 1 year) prior to diagnosis. This lesion was initially recalled by two out of seven readers and only one additional reader converted to recall after reviewing the AI-CAD (neuScore = 75). This case shows potential for more reliance on cmAssist AI-CAD as radiologists gain experience with the software. The magenta box is the cmAssist flag of the right breast mass which corresponds with the validated missed cancer marked by the orange (ground truth) box. The blue box is the cmAssist false flag of calcifications in the left medial breast. AI-CAD, artificial intelligence-based computer-aided detection; CC (CC view only), ‘cranial-caudal’ view; CDR; cancer detection rate; cmAssist, prototype AI-CAD software from CureMetrix, Inc.; LCC, the left ‘cranial-caudal’; LMLO, the left ‘Mediolateral-Oblique’; neuScore, cmAssist quantitative score results (scale of 0–100); RCC, the right ‘cranial-caudal’; RMLO, the left ‘mediolateral-oblique’

It is noted that all readers in this study appeared to ignore relatively significant number of flagged actionable lesions that would have improved their sensitivity even further. This suggests that even further improvement in reader accuracy and CDR could occur as radiologists gain experience in using cmAssist and develop more confidence in its markings and use of the neuScore (quantitative probability of malignancy calculated by cmAssist). While the readers recalled an average of 3.4 additional malignant calcifications cases with benefit of cmAssist, they also disregarded an average of 6.1 flagged malignant calcifications cases. Similarly, while readers recalled an average of 6.4 additional cases of malignant masses with cmAssist assistance, they also disregarded an average of 11.4 cases of flagged malignant mass cases. Figure 4 a and b are an example of a heterogeneously dense breast that shows a large missed cancer. This lesion was initially recalled by two out of seven readers, but only one additional reader converted to recall after reviewing with cmAssist and four of seven readers chose to ignore the flag.

**Fig. 5:**
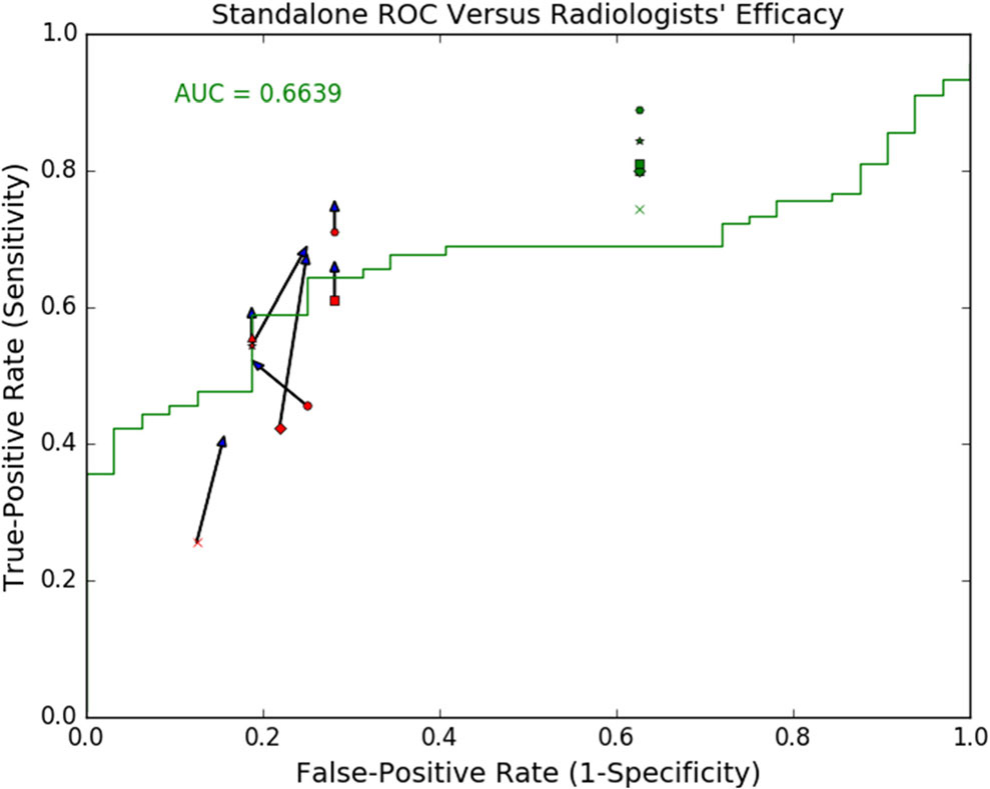
cmAssist AI-CAD stand-alone ROC On 122 Cases. cmAssist’s stand-alone efficacy for the 122 cases in the present study shows an AUC of 0.66. The degraded efficacy compared to Fig. 1 is due to the level of difficulty associated with the 90 cancer cases selected for this study: they represent false-negative cases that had been missed by their interpreting radiologists for up to 5.8 years. The Readers’ efficacy without the assistance of AI-CAD are shown as red markers. The arrows delineate changes in Readers’ efficacy after AI-CAD viewing. The green markers indicate the “theoretical” Readers’ efficacy. With the exception of Reader 5 (Group 2), all readers performed with efficacy subpar of that of AI-CAD. AI-CAD, artificial intelligence-based computer-aided detection; CC (CC view only), ‘cranial-caudal’ view; CDR; cancer detection rate; cmAssist, prototype AI-CAD software from CureMetrix, Inc.; LCC, the left ‘cranial-caudal’; LMLO, the left ‘Mediolateral-Oblique’; neuScore, cmAssist quantitative score results (scale of 0–100); RCC, the right ‘cranial-caudal’; RMLO, the left ‘mediolateral-oblique’

In addition to summarize Tables 1, 2, 3, 4, it is desirable to analyze the data in terms of ROC and AUC. For comparison, cmAssist’s stand-alone ROC for the 122 cases in the present study is shown in Fig. 5, with an AUC of 0.66—noticeably lower than the AUC of 0.900 (95% CI = [0.879, 0.921]) depicted in Fig. 1c, cmAssist stand-alone AUC for all lesion types on the CureMetrix quarantined dataset of 126 biopsy-proven micro-calcification cases, 323 biopsy-proven mass cases, and 836 normal cases (BIRADS 1 and 2 with at least 2 years of follow-up with negative diagnosis). It is noted that the cancer cases chosen for the present study represent false-negative cases that were missed by their original interpreting radiologists, even with the benefit of R2 Imagechecker, for up to 5.8 years, highlighting the level of difficulty of these cases. The reader panel efficacy before viewing cmAssist are shown as red markers in Fig. 5 (each reader is represented by a uniquely shaped marker). Except for Reader 5 (Group 2), all readers performed with efficacy subpar of that of cmAssist.

In Fig. 5, the changes in readers’ efficacy are delineated by the arrows, where the arrowheads mark the readers’ efficacy after viewing cmAssist. The efficacy of all readers, except for Reader 3 (Group 1) and Reader 4 (Group 2), improved above that of stand-alone cmAssist. The green triangles in Fig. 5 represent theoretical readers’ efficacies if no cmAssist flag were ignored. In other words, if the reader did not recall a case before viewing cmAssist, and cmAssist identifies the case with markings, then theoretically, after viewing cmAssist, the reader would change his decision to recall. This rule is applied to normal and biopsy-proven cancer cases alike. Note that the theoretical readers’ efficacies are far above both cmAssist’s stand-alone efficacy and actual readers’ efficacies with cmAssist’s assistance. The results suggest that cmAssist could prove even more beneficial to radiologists than indicated in the present study.

**Fig. 6:**
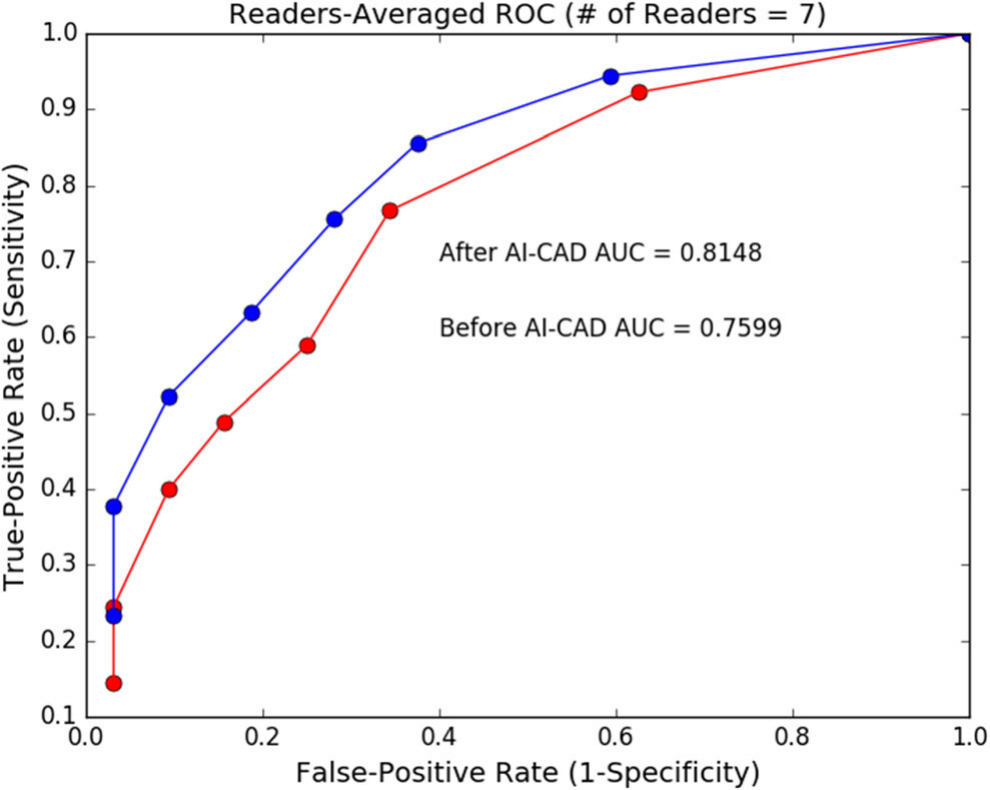
Effect of AI-CAD on readers-averaged ROC. The ROCs before and after review of the AI-CAD results are shown in red and blue, respectively. The AUC for the readers, as a group, is increased from 0.7599 (before AI-ACD) to 0.8148 (after AI-CAD) which represents a 7.2% increase in AUC. AI-CAD, artificial intelligence-based computer-aided detection; CC (CC view only), ‘cranial-caudal’ view; CDR; cancer detection rate; cmAssist, prototype AI-CAD software from CureMetrix, Inc.; LCC, the left ‘cranial-caudal’; LMLO, the left ‘Mediolateral-Oblique’; neuScore, cmAssist quantitative score results (scale of 0–100); RCC, the right ‘cranial-caudal’; RMLO, the left ‘mediolateral-oblique’

Since each reader scores every case with a binary score (0 or 1), it is not possible to construct an ROC for each reader**.** One can, however, compute the ROC for the readers performing as a group. The aggregate score assigned to each case is taken to be the sum of the scores by the 7 readers, and each of the 122 exams has a score between 0 and 7, inclusive. A computation of the ROC for the readers as a group is performed based on their scoring of cases before and after review of the cmAssist results. The ROCs before and after review of the cmAssist results are shown in Fig. 6. The AUC for the readers, as a group, is increased from 0.760 to 0.815 which represents a 7.2% increase in AUC. To assess the statistical significance of the increase in AUC (before versus after cmAssist), we employed the bootstrap analysis [10] in two different ways.

**Fig. 7:**
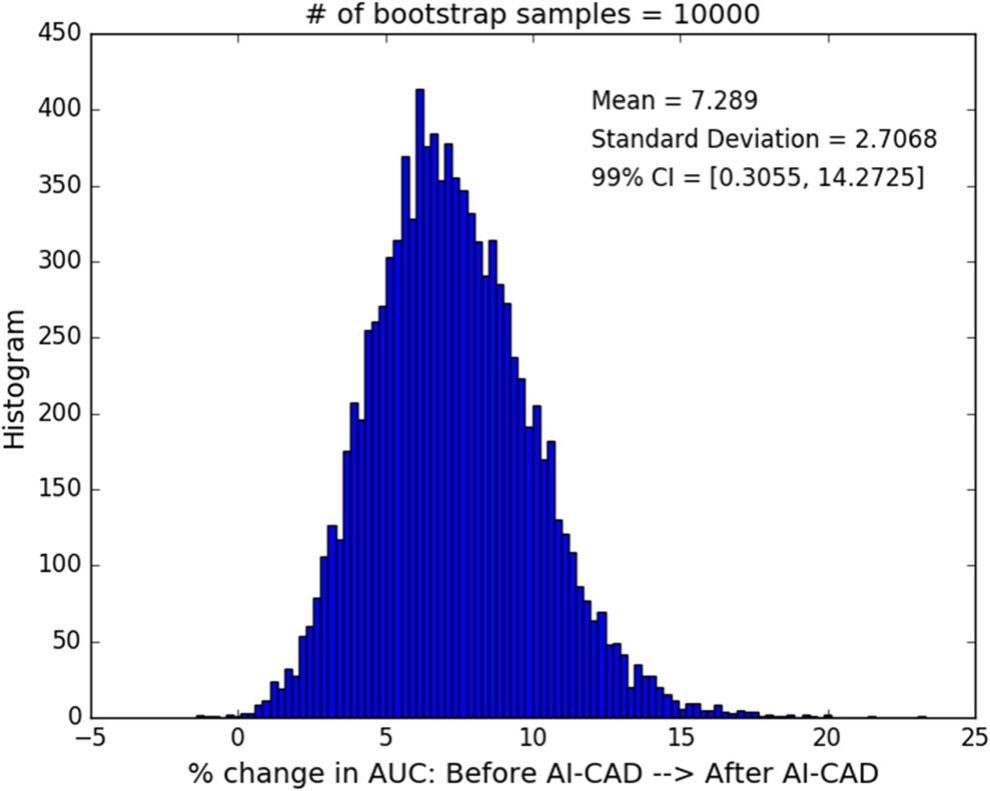
Statistical significance of improvement in readers-averaged AUC with respect to case resampling. A histogram of the percentage change in AUC for the 10,000 bootstrap samples (with respect to the 122 cases in the present study) is shown, with mean *μ* = 7.29% and standard deviation *σ* = 2.71%. The two-sided 99% CI, which corresponds to [*μ* − 2.58*σ*, *μ* + 2.58*σ*] for a normal distribution, is [0.306%, 14.3%]. The null hypothesis of *μ* = 0 is outside of our 99% CI. This test establishes the result’s statistical significance (two-sided *p* < 0.01) with respect to variations in the difficulty level of the cases. AI-CAD, artificial intelligence-based computer-aided detection; CC (CC view only), ‘cranial-caudal’ view; CDR; cancer detection rate; cmAssist, prototype AI-CAD software from CureMetrix, Inc.; LCC, the left ‘cranial-caudal’; LMLO, the left ‘Mediolateral-Oblique’; neuScore, cmAssist quantitative score results (scale of 0–100); RCC, the right ‘cranial-caudal’; RMLO, the left ‘mediolateral-oblique’

First, we resampled with respect to the 122 exams, using 10,000 bootstrap samples. For each given bootstrap sample, we computed the readers-averaged ROCs before and after review of the cmAssist results. From the resulting ROCs, the change in AUC is computed for the bootstrap sample. A histogram of the percentage change in AUC, defined as 100 × (before-cmAssist AUC − after-cmAssist AUC)/before-cmAssist AUC, is shown in Fig. 7. To formalize the statistical significance, we compute the two-sided 99% confidence interval (CI) for the percentage change in AUC ([0.306%, 14.3%]), and the null hypothesis (mean change in AUC = 0) is outside of our 99% CI. This test establishes the results’ statistical significance (*p* < 0.01) with respect to variations in the difficulty level of the cases.

**Fig. 8:**
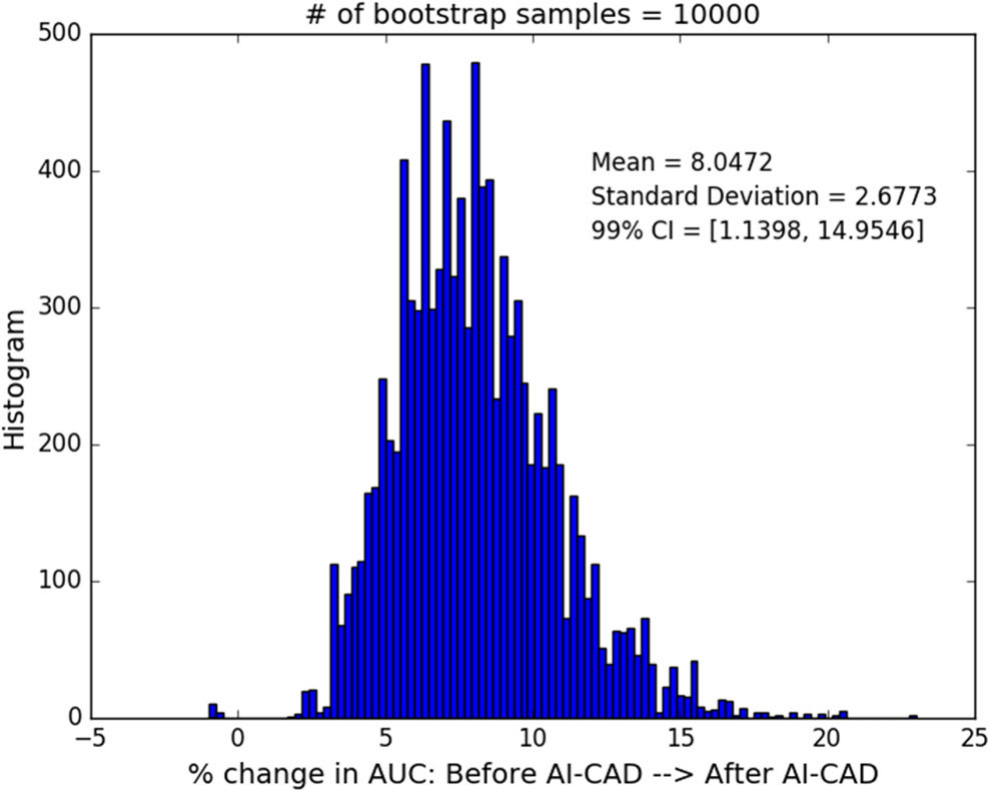
Statistical significance of improvement in readers-averaged AUC with respect to reader resampling. A histogram of the percentage change in AUC for the 10,000 bootstrap samples (with respect to the 7 Readers in the present study) is shown, with mean *μ* = 8.05% and standard deviation *σ* = 2.68%. The two-sided 99% CI is [1.14%, 15.0%]. The null hypothesis of *μ* = 0 is outside of our 99% CI. This test establishes the result’s statistical significance (two-sided *p* < 0.01) with respect to variations in the difficulty level of the cases. AI-CAD, artificial intelligence-based computer-aided detection; CC (CC view only), ‘cranial-caudal’ view; CDR; cancer detection rate; cmAssist, prototype AI-CAD software from CureMetrix, Inc.; LCC, the left ‘cranial-caudal’; LMLO, the left ‘Mediolateral-Oblique’; neuScore, cmAssist quantitative score results (scale of 0–100); RCC, the right ‘cranial-caudal’; RMLO, the left ‘mediolateral-oblique’

Similarly, we perform the same analysis, also using 10,000 bootstrap samples, but we resample with respect to the readers to assess statistical significance with respect to variations in the readers’ experience level, and a histogram of the percentage change in AUC is shown in Fig. 8. Once again, the null hypothesis (mean change in AUC = 0) is outside of our two-sided 99% CI ([1.14%, 15.0%]), and the results’ statistical significance with respect to the variations in the readers’ experience level is established with *p* < 0.01.

## Discussion

Retrospective findings may be seen on prior mammograms of breast cancer patients 52 to 75.3% of time [11]. This rate is higher than our results where 44% of breast cancer patients had retrospective findings. Therefore, the number of retrospective findings in this study does not represent a lack of skill on the part of the original interpreting radiologists and instead represents a potential area for improvement for all radiologists. This study addresses the potential for early detection of breast cancers using AI. The cmAssist AI-CAD flagged missed malignant lesions in this dataset of prior studies as early as 70 months (5.8 years) prior to recall or diagnostic workup.

Variability in breast imaging radiologists’ performance is well recognized and has been widely reported, first by Beam and Sullivan [12] and, more recently, by the Breast Cancer Surveillance Consortium [9]. The variability in sensitivity of the readers is also reflected in this study and appears to correspond with differences in training and experience.

One of the trends seen in this study was that radiologists with the least experience and training derived the most improvement in performance with cmAssist, consistent with previous works [13]. The fellowship-trained mammographers and an experienced general radiologist showed the least improvement in CDR with cmAssist. This is in part due to the higher CDR achieved by these experienced readers on their own, without cmAssist. However, this may also reflect the group’s negative bias to disregard CAD flags due to personal experience with currently available CAD systems. The experienced mammography-trained radiologists (Group 2) ignored more of the flagged actionable lesions than the less-experienced radiologists (Group 1), as shown in Table 1. Similarly, except for Reader 4, readers in group 2 did not reverse any of their false-positive recalls, as shown in Table 2. The dramatic improvement in CDRs for the recently trained general radiologists in this study suggests a greater acceptance of new AI technologies by younger, more technologically adept physicians who may be less confident in their mammography skills.

Results indicate that the sensitivity of all readers in this study appeared to be elevated due to the test setting and the enrichment of the data set with a high proportion of abnormal mammograms. This “laboratory effect” has been described, among others, by Gur et al. [14]. These factors also account for false-positive recall rates that are higher than would occur in clinical practice.

In this study, the radiologist panel had a slightly greater tendency to disregard flags of actionable masses over calcifications. Even the most accurate reader in the study ignored 18% of the cmAssist flagged Actionable lesions. The improvement in CDRs could have been much higher if the readers relied more on the cmAssist results for decision-making, as shown in Fig. 5. The primary focus of this paper was to determine whether the use of AI-enabled CAD can increase the sensitivity of radiologists in a dataset enriched with biopsy-proven cancer cases and false-negative cancer cases that had been missed on their initial reading. Thus, the study is reported using CDR in addition to ROC in assessing effect on radiologist interpretations.

**Fig. 9:**
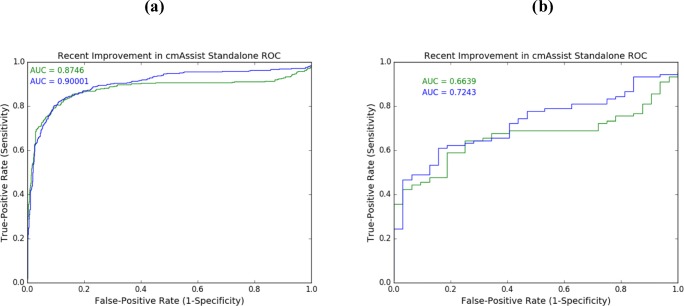
Recent improvement in cmAssist stand-alone efficacy. cmAssist’s stand-alone combined efficacy (for both micro-calcifications and masses), based on two separate datasets are shown: **a** the CureMetrix quarantined test dataset consisting of 836 normal studies, 126 biopsy-proven micro-calcification cancer studies, and 323 biopsy-proven mass cancer studies, and **b** the 122 cases in the present study (90 biopsy-proven cancer studies and 32 normal studies). The green and blue curves represent the cmAssist efficacies at the time of the Readers study and at the time of current writing, respectively. AI-CAD, artificial intelligence-based computer-aided detection; CC (CC view only), ‘cranial-caudal’ view; CDR; cancer detection rate; cmAssist, prototype AI-CAD software from CureMetrix, Inc.; LCC, the left ‘cranial-caudal’; LMLO, the left ‘Mediolateral-Oblique’; neuScore, cmAssist quantitative score results (scale of 0–100); RCC, the right ‘cranial-caudal’; RMLO, the left ‘mediolateral-oblique’

In the short span between the completion of the reader study reported here and the time of current writing, substantial progress has been made on the stand-alone efficacy of cmAssist, as evidenced in Fig. 9 a and b where the green and blue curves denote the ROC for cmAssist at the time of the reader study and at the time of current writing, respectively. The improvement in the cmAssist algorithm resulted in a 9% increase in AUC for the dataset used in the reader study.

It is again emphasized that the test cases in this study have never been provided as training cases in the development of cmAssist.

There will be continued improvement in accuracy as algorithms are refined, more training data is included, and computer processing power increases. It should be noted that the maximum achievable sensitivity for cmAssist in stand-alone mode on this data set of missed cancers was 98%. Future work in false-positive reduction using AI is in progress and will lead to further improvements in accuracy for cancer detection. With the advent of 3D tomosynthesis, there is a reported further improvement in accuracy over 2D digital mammography, but with cost of higher radiation dose and expense. There has been partial clinical implementation of 3D tomosynthesis in the USA and Western Europe which is expected to grow. Most facilities still perform 2D mammography views in conjunction with 3D in part due to higher conspicuity of calcifications but also related to issues with reader confidence, archiving and comparing with prior studies. It is anticipated that the principles of AI-CAD used in development of cmAssist will translate to 3D and synthetic 2D mammograms as well.

This analysis of the false-positive recall rate in this study is limited due to the relative small sample size of normal cases. However, it would not have been reasonably possible to reflect the true prevalence of malignancy in a study of this nature. Another limitation of this reader study is the lack of comparison of prior mammograms, which could have resulted in increased reader sensitivity [14]. Because this was a study with enriched data consisting of primarily cancer cases, the authors could not assess specificity accurately. CDR was used for the main analysis in this study in addition to standard ROC because this test set had an unusually high number of cancer cases compared to usual percentage seen clinically in a general screening population.

This study shows how AI-based software can provide clinical benefit to radiologists in interpretation of screening mammograms. The use of AI in clinical practice may potentially expedite workflow, enhance earlier detection of cancer, and reduce false-negative mammograms. The impact of AI on medical imaging in the future is likely to be profound. To the authors’ knowledge, this is the first peer-reviewed scientific study that shows significant benefit of AI to radiologists in clinical image interpretation.

## Electronic supplementary material

ESM 1 (DOCX 18 kb)

## Abbreviations

2-D: Two-dimensional
AI: Artificial intelligence
AI-CAD: Artificial intelligence-based computer-aided detection
BIRADS: Breast imaging and reporting data system
CAD: Computer-aided detection
CDR: Cancer detection rate
FFDM: Full-field digital mammograms
HIPAA: Health insurance portability and accountability act
IRB: Institutional review board
MQSA: Mammography Quality Standards Act. MQSA standards (i.e., temperature, ambient light, light sources [less than 50 lx], level of comfort, type of furnishings including monitors, and ambient noise)
