# Supplementary material for: Improved Cancer Detection Using Artificial Intelligence: a Retrospective Evaluation of Missed Cancers on Mammography
Source: J Digit Imaging. 2019 Apr 22;32(4):625–37. doi: 10.1007/s10278-019-00192-5 (PMC6646649; doi:10.1007/s10278-019-00192-5)
Supplement: Supplementary file 1 — (DOCX 18 kb) [file 10278_2019_192_MOESM1_ESM.docx]

**Supplemental Materials**

Supplemental table which is not referenced in the manuscript due to guideline restrictions but provided for reviewers.

## **Supplementary Table 1: Actionable Lesions on False-Negative Mammograms**

| **Supplementary Table 1: Actionable Lesions on False Negative Mammograms** | |
| --- | --- |
| **Lesion Type** | **Count** |
| **Mass** | 50 |
| **Microcalcifications** | 16 |
| **Mass and Microcalcifications** | 9 |
| **Architectural Distortions** | 5 |
| **Mass and Architectural Distortions** | 4 |
| **Asymmetry** | 3 |
| **Architectural Distortion and Microcalcifications** | 1 |
| **Microcalcifications and Asymmetry** | 1 |
| **Focal Asymmetry** | 1 |
| **Total** | 90 |

**Supplementary Table 1:** summarizes the Actionable lesion types on the false negative mammograms as defined by the validating radiologists. For brevity, all cases with microcalcifications as the leading lesion type are categorized as calcifications and all remaining cases as mass. The Actionable prior mammogram cases consisted of 17 calcification cases and 73 mass cases.
